# Genome-wide gene responses in a transgenic rice line carrying the maize resistance gene *Rxo1 *to the rice bacterial streak pathogen, *Xanthomonas oryzae *pv. *oryzicola*

**DOI:** 10.1186/1471-2164-11-78

**Published:** 2010-02-01

**Authors:** Yong-Li Zhou, Mei-Rong Xu, Ming-Fu Zhao, Xue-Wen Xie, Ling-Hua Zhu, Bin-Ying Fu, Zhi-Kang Li

**Affiliations:** 1 Institute of Crop Sciences/National Key Facility for Crop Gene Resources and Genetic Improvement, Chinese Academy of Agricultural Sciences, 12 South Zhong-Guan-Cun St., Beijing 100081, PR China; 2 Fujian Academy of Agricultural Sciences, Fu Zhou, 350003, PR China; 3 International Rice Research Institute, DAPO Box 7777, Metro Manila, the Philippines

## Abstract

**Background:**

Non-host resistance in rice to its bacterial pathogen, *Xanthomonas oryzae *pv. *oryzicola *(*Xoc*), mediated by a maize NBS-LRR type R gene, *Rxo1 *shows a typical hypersensitive reaction (HR) phenotype, but the molecular mechanism(s) underlying this type of non-host resistance remain largely unknown.

**Results:**

A microarray experiment was performed to reveal the molecular mechanisms underlying HR of rice to *Xoc *mediated by *Rxo1 *using a pair of transgenic and non-transgenic rice lines. Our results indicated that *Rxo1 *appeared to function in the very early step of the interaction between rice and *Xoc*, and could specifically activate large numbers of genes involved in signaling pathways leading to HR and some basal defensive pathways such as SA and ET pathways. In the former case, *Rxo1 *appeared to differ from the typical host R genes in that it could lead to HR without activating *NDR1*. In the latter cases, *Rxo1 *was able to induce a unique group of WRKY TF genes and a large set of genes encoding PPR and RRM proteins that share the same G-box in their promoter regions with possible functions in post-transcriptional regulation.

**Conclusions:**

In conclusion, *Rxo1*, like most host *R *genes, was able to trigger HR against *Xoc *in the heterologous rice plants by activating multiple defensive pathways related to HR, providing useful information on the evolution of plant resistance genes. Maize non-host resistance gene *Rxo1 *could trigger the pathogen-specific HR in heterologous rice, and ultimately leading to a localized programmed cell death which exhibits the characteristics consistent with those mediated by host resistance genes, but a number of genes encoding pentatricopeptide repeat and RNA recognition motif protein were found specifically up-regulated in the *Rxo1 *mediated disease resistance. These results add to our understanding the evolution of plant resistance genes.

## Background

Within the natural environments, crop plants are continuously confronted with different potential pathogens and pests because of their sessile characteristics. As a result, they have evolved complicated defense mechanisms to protect themselves from these pathogenic microorganisms. These include hypersensitive reaction (HR, which is characterized by the rapid cell death surrounding the infection sites), increased expression of defense related genes, and the oxidative burst [1]. The well-studied plant defense mechanism is the host resistance mediated by the products of plant resistance (R) genes. Up to date, large numbers of R genes have been identified and isolated from major crop species such as wheat (*Triticum aestivum*), rice (*Oryza sativa*) and maize (*Zea mays*) [2]. The majority of these plant R genes encode proteins containing a central domain with a nucleotide binding site (NBS) and a carboxyterminal domain consisting of a series of degenerated leucine-rich repeat (LRR) residues. All of the isolated crop R genes reported so far confer resistance to a narrow spectrum of resistance to a small number of pathogen races in a gene-for-gene manner. This kind of race specific resistance is normally short-lived in agricultural utilization resulting from either loss or alteration of avirulence gene products in the fast evolving pathogens [3].

Although highly effective in controlling many diseases, host resistance mediated by R genes does not exist in some other cases in any specific host plant species. For example, *Xanthomonas oryzae *pv. *oryzae *(*Xoo*) and *Xanthomonas oryzae *pv. *oryzicola *(*Xoc*) are two highly related pathogens of rice (*Oryza sativa*L) that constrain rice production in large rice growing areas of Asia and Africa. The former causes bacterial blight by invading rice vascular tissues and the latter causes bacterial leaf streak by colonizing plant parenchyma. Tremendous progress has been made in breeding for resistance to *Xoo *in rice by using one or more of the identified 30 R genes [4]. To *Xoc*, however, no resistance controlled by single R genes has been identified in rice, even though a few quantitative trait loci for resistance to bacterial leaf streak have been reported [5].

Non-host resistance refers to the immune responses of all members of a plant species to all members of a given pathogen species [6,7]. There have been increasing interests in non-host resistance because of its potential use in disease control of heterologous plant species. A successful case in this area was the cloning of a maize R gene, *Rxo1 *and demonstration of its function in conferring HR to *Xoc *in transgenic rice [8,9]. *Rxo1 *is a dominant NBS-LRR type *R *gene identified in maize. The transgenic rice lines with cloned *Rxo1 *exhibited a distinct HR symptom when inoculated with *Xoc *[10,11], demonstrating that certain *R *genes can be effectively transferred between distantly related cereals.

Although significant progress has been made in understanding the molecular mechanisms of R gene mediated defenses against pathogens in plants, much less is known about the non-host R gene- pathogen defense responses in heterologous plants. In this paper, we report the use of the Affymetrix GeneChip system to identify genes that are differentially regulated during incompatible and compatible interactions between *Rxo1 *and *Xoc *in a near isogenic pair of transgenic and non-transgenic lines in the 9804 background. Our results elucidated some interesting molecular mechanisms underlying rice resistance to *Xoc *mediated by the non-host NBS-LRR maize R gene, *Rxo1*.

## Results

### Phenotypic reactions of 9804-Rxo1 and 9804 to Xoc

**Figure 1:**
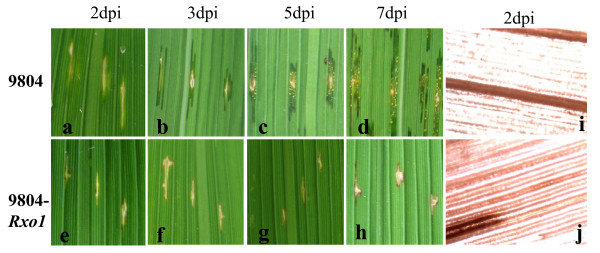
**Different responses of 9804-Rxo1 and 9804 to Xanthomonsa oryzae pv. oryzicola**. Left: phenotypes; Right: lignin histochemical staining. Dpi: days post inoculation. a, b, c and d are the phenotypes of 9804 at 2, 3, 5 and 7 dpi; e, f, g and h are the phenotypes of 9804-Rxo1 at 2,3,5 and 7 dpi; and i and j are the microscopy images of lignin histochemical staining of leaves of 9804 and 9804-Rxo1 at 2 dpi, respectively.

A susceptible chlorotic symptom was visible as soon as 2 days post-inoculation (dpi) on the inoculated flag leaves of 9804 plants, and then the water-soaked lesions began to spread along the pricked sites 3 dpi (Fig. 1a, b). At 5 dpi, 9804 had an average lesion length of 4.93 ± 0.45 cm with masses of bacteria accumulated on the lesion surfaces (Fig. 1c). The lesions expanded further on the inoculated leaves of 9804 7 dpi (Fig. 1d). In contrast, the transgenic 9804-*Rxo1 *plants were resistant to *Xoc*, showing typical lightly brown edges around the pricked sites and restricted necrotic lesions on the inoculated leaves 3 dpi (Fig. 1e, f) with an average lesion length of 0.41 ± 0.11 cm at 5 dpi. The resistance phenotype of the 9804-*Rxo1 *was more apparent at 7 dpi with the typical ring-shaped necrosis surrounding the infection sites (Fig. 1g, h). Lignin histochemical staining revealed that when compared with susceptible 9804, lignin deposition occurred to a great extent in the distinctly thickened hydathodes and xylem walls of the infected leaves of the resistant 9804-*Rxo1 *plants within 48 h (Fig. 1i, j), an indication of HR [12,13].

### Quantitative differences in gene expression between 9804-Rxo1 and 9804 after in response to inoculation with Xoc

We detected 2450 and 1950 differentially regulated genes (DRGs) in 9804-*Rxo1 *and 9804 2 dpi using a combined criterion of more than two-fold change in expression and a *P *value < 0.05 in *t *tests. The difference between 9804-*Rxo1 *and 9804 in expression patterns of these up- or down-regulated genes was more striking. Of the 1239 and 963 up-regulated genes in 9804-*Rxo1 *and 9804 induced by *Xoc*, only 143 genes were in common between the transgenic line and its recipient. Similarly, of the 1211 and 987 down-regulated genes in 9804-*Rxo1 *and 9804 induced by *Xoc*, 83 genes were commonly repressed in both the transgenic line and its recipient [Additional file 1]. In particular, we detected 107 genes which were regulated in opposite directions between 9804-*Rxo1 *and 9804, including 61 genes up-regulated in 9804-*Rxo1 *but down-regulated in 9804 and 46 genes down-regulated in 9804-*Rxo1 *but up-regulated in 9804, respectively.

**Figure 2:**
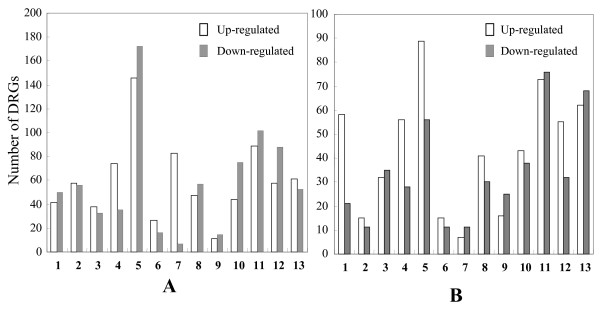
**The numbers of differentially regulated genes (DRGs) in different function categories in 9804-Rxo1 (A) and 9804 (B) under the infection of Xanthomonas oryzae pv. oryzicola respectively**. Only those genes with known or putative functions were included. The numbers on the X axis indicate different function categories: **1 **abiotic stress response; **2 **cellular processes and cytoskeleton; **3 **cell wall and membrane; **4 **defense response and disease resistance; **5 **metabolism; **6 **phytohormone regulation; **7 **post translation modification; **8 **redox regulation; **9 **secondary metabolism; **10 **transcription factor; **11 **transcription regulation and signal transduction; **12 **transport; **13 **Others.

Fig. 2 shows all DRGs classified into different functional categories according to Gene Ontology analysis using FatiGO http://fatigo.bioinfo.cipf.es combined with UniProt http://www.uniprot.org identified in 9804-*Rxo1 *and 9804. 9804-*Rxo1 *had many more genes involved in cellular processes and post translation modification significantly up-regulated, while most up-regulated genes in 9804 belong to the function categories of abiotic stress response, defense response and disease resistance, metabolism and transport (Fig. 2). Interestingly, of the small number of DRGs that were commonly regulated in 9804-*Rxo1 *and 9804 [Additional file 1], the up-regulated genes belong to functional groups of abiotic stress responses, cellular process and cell wall biogenesis, defense responses, disease resistance and redox regulation, while the down-regulated ones were mainly involved in transcription regulation and signal transduction.

**Figure 3:**
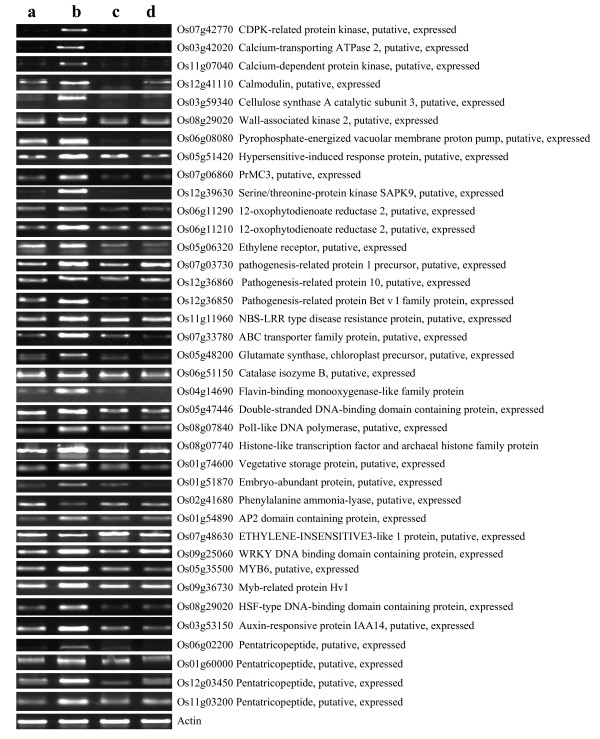
**The RT-PCR profiles of 38 selected differentially regulated genes induced by 2 dayspost-inoculation of Xanthomonas oryzae pv. oryzicola**. Letters **a, b, c **and **d **represent the leaf samples from the non-inoculated 9804-Rxo1, inoculated 9804-Rxo1, non-inoculated 9804 and inoculated 9804, respectively.

Fig. 3 shows the RT-PCR profiles of a selected group of 38 DRGs of different categories identified in the microarray experiment. The RT-PCR results confirmed the microarray results with regard to the expression patterns of all selected genes in the infected leaves of 9804-*Rxo1 *and 9804.

### Differential expression of transcription factors (TF) in 9804-Rxo1 and 9804

The infection of *Xoc *resulted in diferential expression of 121 and 83 TF genes in most TF families (WRKY, MYB, bZIP, AP2, NAC, zinc finger, bHLH, GRAS, and MADS) in 9804-*Rxo1 *and 9804 [Additional file 2, 3], but most of differentially expressed TFs were different in 9804-*Rxo1 *and 9804 except for six TF genes (*OsWRKY23*, *OsWRKY45*, *OsWRKY76*, *ANAC071*, and *RING113A *induced and *OsIAA21 *repressed). In addition, three TFs (*OsNAC77*, *OsWRKY28 *and one ZIM TF) showed opposite expression patterns in 9804-*Rxo1 *and 9804 in response to the infection of *Xoc*.

Of these differentially regulated TFs detected, we detected 9 and 8 TF genes of the WRKY family in 9804-*Rxo1 *and 9804, respectively (Table 1), of these three genes, *OsWRKY23 *(Os01g53260), *OsWRKY45 *(Os05g25770) and *OsWRKY76 *(Os09g25060) were commonly induced by *Xoc *in both lines. One gene, *OsWRKY28 *(Os06g44010) was significantly down-regulated in 9804-*Rxo1*, but highly up-regulated in 9804. This gene plus the remaining 9 differentially regulated WRKY TFs, including 5 (Os01g18584, Os12g02440, Os11g02540, Os05g03900, Os01g14440) detected in 9804-*Rxo1 *and 4 (Os05g49620, Os11g29870, Os05g50610, Os01g60540) in 9804 may have played an important role in mediating the resistance reaction of 9804-*Rxo1 *to *Xoc*.

**Table 1:** Twenty-nine differentially regulated transcription factor genes in the 9804-*Rxo1 *and 9804 plants infected by *Xoc*

NCBI Code	9804-*Rxo1*	9804	Transcript Assignments
Os10g42130.1	2.96	8.07	ANAC071, putative, expressed
Os01g01470.1	0.22	20.25	NAC domain-containing protein 77, putative, expressed/
Os03g08330.1	0.43	2.31	ZIM motif family protein, expressed
Os07g47790.1		3.58	ERF-like protein, putative, expressed
Os07g22730.1		2.97	ethylene-responsive transcription factor 15, putative, expressed
Os06g07040	0.49		AUX/IAA family protein, expressed
Os11g32110.	0.45		Auxin response factor 2, putative, expressed
Os06g22870.1	0.26	0.46	OsIAA21 - Auxin-responsive Aux/IAA gene family member
Os03g58350.1		0.43	OsIAA14 - Auxin-responsive Aux/IAA gene family member, expressed
Os06g39590.1		0.41	OsIAA23 - Auxin-responsive Aux/IAA gene family member, expressed
Os12g40890.1		0.15	OsIAA30 - Auxin-responsive Aux/IAA gene family member, expressed
Os02g49160.1		0.23	OsIAA8 - Auxin-responsive Aux/IAA gene family member, expressed
Os07g29310.1		0.35	OsSAUR30 - Auxin-responsive SAUR gene family member, expressed
Os12g41600.1		0.17	OsSAUR57 - Auxin-responsive SAUR gene family member, expressed
Os03g22450.1	2.27		auxin response factor 75, putative, expressed
Os01g18584.1	0.43		OsWRKY9 - Superfamily of rice TFs having WRKY and zinc finger domains, expressed
Os05g03900	0.49		WRKY DNA binding domain containing protein, expressed
Os11g02540	0.32		WRKY DNA binding domain containing protein, expressed
Os12g02440	0.12		WRKY DNA binding domain containing protein, expressed
Os01g14440	2.33		WRKY DNA binding domain containing protein, expressed
Os01g60540.1		0.38	OsWRKY20 - Superfamily of rice TFs having WRKY and zinc finger domains
Os05g49620.1		2.07	OsWRKY19 - Superfamily of rice TFs having WRKY and zinc finger domains, expressed
Os11g29870.1		3.80	OsWRKY72 - Superfamily of rice TFs having WRKY and zinc finger domains, expressed
Os05g50610.2		3.56	OsWRKY8v2 - Superfamily of rice TFs having WRKY and zinc finger domains, expressed
Os01g53260.1	2.39	2.09	OsWRKY23 - Superfamily of rice TFs having WRKY and zinc finger domains, expressed
Os05g25770.1	2.77	2.40	OsWRKY45 - Superfamily of rice TFs having WRKY and zinc finger domains, expressed
Os09g25060.1	4.90	2.46	OsWRKY76 - Superfamily of rice TFs having WRKY and zinc finger domains, expressed
Os02g19804.1	2.49	2.58	RING finger protein 113A, putative, expressed/
Os06g44010.1	0.48	7.36	OsWRKY28 - Superfamily of rice TFs having WRKY and zinc finger domains, expressed

The second group of interesting TF genes included several IAA responsive genes that were significantly repressed in either or both lines under the infection of *Xoc *(Table 1), plus *OsARF75 *(Os03g22450) which was induced only in 9804-*Rxo1*. The former group included *OsIAA21 *which was down-regulated by *Xoc *in both lines; Os06g07040 and *OsARF2 *that were repressed only in 9804-*Rxo1*, and six other genes (*OsIAA8*, *OsIAA14*, *OsIAA23*, *OsIAA30*, *OsSAUR57*, *and OsSAUR30*) that were repressed only in 9804. Distinct expression patterns of these auxin responsive genes 9804-*Rxo1 *and 9804 indicated that they may have played important roles in both resistant and susceptible reactions of rice to *Xoc*.

### Specific groups of genes possibly related to post-transcription regulation in the Rxo1 mediated resistance to Xoc reaction

We detected several other groups of DRGs in this study that might be related to *Rxo1 *mediated resistance in rice to *Xoc*. The first group included 82 DRGs functionally classified as post-transcription regulation. Of these, 68 putative genes encoding pentatricopeptide repeat (PPR) were differentially regulated specifically in the infected 9804-*Rxo *1 plants, and strikingly, 65 of them were significantly up-regulated. An additional group of 8 genes encoding RNA recognition motif (RRM) proteins were all highly induced in 9804-*Rxo1 *under the *Xoc *infection [Additional file 4].

To identify the common *cis*-elements of the DRGs coding for PPR, we searched the *cis*-regulatory elements in the 1 kb regions upstream of these DRGs using WeederWin1.3 software. Three unique sequences: AACTGGAC, GAAACTGG and AACTGG, were found specifically enriched in the upstream of 65 up-regulated PPR genes (Additional file 5). In most cases, the three motifs locate between -10 to -1000 bp upstream of the ATG start codon of the PPR genes. Further analyses revealed a G-box, AACTGG, within the cis-element in the promoter fragments of these genes, which is known as the binding (recognition) site to a JA-responsive MYC2 transcription factor [14], indicating that these PPR genes may have been involved in the JA mediated disease responses.

### DRGs involved in HR in 9804-Rxo1 under the infection of Xoc

Because 9804-*Rxo1 *showed the same HR phenotype to the infection of *Xoc *as that mediated by most R genes in host plants, we expect that *Rxo1 *must be able to trig expression of genes involved in HR. Indeed, large numbers of genes involved in calcium ion fluxes and oxidative burst were uniformly up-regulated in the infected 9804-*Rxo1 *plants compared with those in 9804 of our microarray data (Table 2 and Additional file 6). Specifically, 4 genes associated with calcium ion fluxes were significantly up-regulated in 9804-*Rxo1*, including calcium-transporting ATPase 2 (Os03g42020), calcium-dependent protein kinase (Os11g07040, Os07g42770), and Calmodulin (Os12g41110). The expression patterns of the 3 genes were further confirmed by RT-PCR (Fig. 3). Four other genes associated with the accumulation of reactive oxygen species during HR were specially up-regulated in 9804-*Rxo1*, including amine oxidase (Os05g48200), vacuolar H^+^-pyrophosphatase (Os06g08080), respiratory burst oxidase 2 (Os01g25820), and flavin-containing monooxygenase family protein (Os04g14690).

**Table 2:** Twenty-five genes related to PCD up-regulated specially in the leaves of 9804-*Rxo1 *infected by *Xoc *2 days post-inoculation

NCBI Code	9804-*Rxo1*	9804	Transcript Assignments
Os06g06400	2.98		NB-ARC domain containing protein
Os07g33690	2.69		NB-ARC domain containing protein
Os06g063800	2.52		NB-ARC domain containing protein
Os01g52340	2.22		NB-ARC domain containing protein
Os09g34150	2.14		NB-ARC domain containing protein
Os11g12330	2.11		NB-ARC domain containing protein
Os05g31570	0.49		NB-ARC domain containing protein
Os03g14900	0.44		NB-ARC domain containing protein
Os01g52270	0.40	2.09	NB-ARC domain containing protein
Os09g10054	0.33		NB-ARC domain containing protein
Os03g16780	15.21		Ankyrin repeat family protein
Os06g13000	2.88		Ankyrin repeat protein
Os01g61990	2.13		Ankyrin repeat family protein
Os09g24900	2.02		Ankyrin protein kinase
Os03g59090	0.48		Cell death suppressor protein Lls1 homolog
Os09g03680	2.09		Death-associated protein kinase 1
Os01g06740	3.60		Ribosome inactivating protein
Os12g17540	2.87	0.5	Vignain precursor
Os04g31030	3.29		Nitrate-induced NOI protein
Os10g41534	2.35		ICE-like protease p20 domain containing protein
Os10g07010	2.25	4.05	Senescence-associated protein 15
Os02g49630	0.43	0.33	Senescence-associated protein 5
Os07g06860	5.49		PrMC3
Os06g04460	2.29		hypersensitive-induced reaction protein 4
Os05g51420	4.21		hypersensitive-induced response protein

A third group of 25 genes possibly associated with PCD were differentially expressed in infected 9804-*Rxo1 *leaves (Table 2). Of these 19 were significantly up-regulated, including genes of the NB-ARC domain, ankyrin repeat protein ACD6, and genes encoding proteins of the Ankyrin repeat family. The NB-ARC domain is a novel protein motif shared by many important plant and animal proteins which activate cell death [15]. Table 2 shows 10 NB-ARC domain containing protein genes that were differentially expressed in the infected leaves of 9804-*Rxo1*, 6 of which were significantly up-regulated.

Inoculation of 9804-*Rxo1 *with *Xoc *also specifically up-regulated 9 genes, including four ankyrin repeat family proteins or ankyrin protein kinases genes (Os03g16780, Os06g13000, Os01g61990 and Os09g24900), two genes (Os05g51420 and Os07g06860) of putative hypersensitive-induced response proteins, a protein homologue PrMC3 (Os07g06860, a 2-hydroxyisoflavanone dehydratase), the death-associated protein kinase 1 (Os09g03680) and a ribosome inactivating protein (RIP) gene (Os01g06740), and repressed a gene encoding the cell death suppressor protein Lls1 homolog (*Os Lls1*, Os03g59090). All these genes were previously reported to play key roles in the hypersensitive cell death reaction, biotic and abiotic stress-related biological processes [16-20]. The expression patterns of (Os05g51420 and Os07g06860) were confirmed by RT-PCR (Fig. 3). We noted that only 2 HR related genes (Os10g07010 and Os02g49630) showed the same expression patterns in both 9804-*Rxo1 *and 9804. The former was up-regulated in both lines and the latter was down-regulated in both lines (Table 2).

### DRGs involved in SA/JA/ET signaling pathways

**Figure 4:**
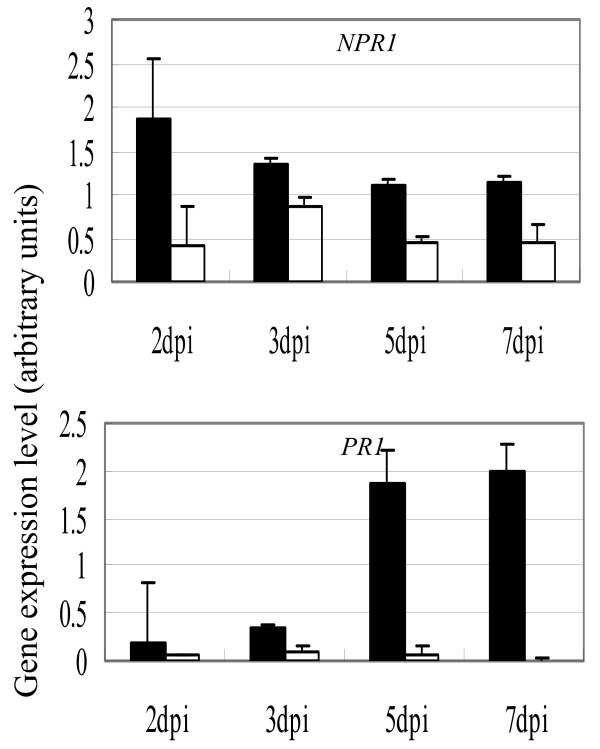
**The timing expression patterns of OsNPR1 (Os03g46440) and OsPR1 (Os07g03730) in 9804-Rxo1 under the infection of Xoc**. The gene expression level was quantified by real-time PCR at 2, 3, 5 and 7 dpi. Closed bars represent resistant 9804-Rxo1/Xoc; Open bars represent 9804/Xoc. Gene expression level (arbitrary units) was normalized using actin as an internal reference.

Inoculation of *Xoc *resulted in identification of several DRGs involved in SA-, JA- and ET-dependent signaling pathways in 9804-*Rxo1 *and 9804. For example, two genes, *OsNPR1 *(Os03g46440) and *OsWRKY62 *(Os09g25070) involved in the SA pathway [21,22], were specifically up-regulated in 9804-*Rxo1*. Three additional SA-responsive *PR *genes encoding pathogenesis-related protein 1 precursor (Os*PR1*, Os07g03730), PR protein 10 (*OsPR10*, Os12g36860) and PR Bet/v1 family protein (Os12g36850) were 11.1-, 5.8- and 4.1-fold up-regulated in the infected 9804-*Rxo1 *(Fig. 3). In contrast, the expression of the five genes didn't show any change in the *Xoc *infected 9804 plants. We further examined the timing expression patterns of Os*NPR1 *(Os03g46440) and Os*PR1 *precursor (Os07g03730) in 9804-*Rxo1 *using real-time PCR. These two genes were induced in both 9804-*Rxo1*and 9804 at 2 dpi, but the expression level of the two genes were much higher in the infected 9804-*Rxo1 *plants than that in the infected 9804 (Fig. 4) and increased gradually in the infected 9804-*Rxo1 *from 2 dpi to 7 dpi. In contrast, the expression of *OsNPR1 *and *OsPR1 *genes in the infected 9804 plants remained relatively stable at a very low level.

In addition, several DRGs related to the JA and ET signaling pathways were also identified. These included three genes encoding ethylene-responsive factor-like protein 1 (OsERF1, Os01g21120), ethylene-responsive transcription factor 2 (OsERF2, Os01g54890), ethylene receptor (Os05g06320), which were highly (14.5-, 2.89- and 3.2-fold) up-regulated in the 9804-*Rxo1*plants by *Xoc*. However, a small set of genes related to ethylene biosynthesis (Os04g10350, Os04g48850, Os05g10780, and Os02g43790) and ethylene signaling pathway (*OsERF5*, Os07g22730) were detected up-regulated only in the infected 9804 plants. Two additional genes (Os12g14440, Os12g12720) encoding jasmonate-induced protein functioning in the JA signaling pathway were significantly up-regulated by the *Xoc *attack in both rice lines.

## Discussion

Non-host resistance is rarely studied since this form of resistance is generally thought to be genetically complex and the activation of any specific defense component may not be sufficient to render a plant resistance reaction [23,24]. There is growing evidence that single non-host R genes can activate complex defense processes such as HR in heterologous host plants in response to specific host pathogens, resulting in similar resistance phenotype governed by host R genes [[8,9,25], and [26]]. However, this process remains poorly understood at the molecular level. To answer this question, we analyzed the genome-wide responses in gene expression of the transgenic rice line carrying a non-host maize R gene, 9804-*Rxo1 *and its recipient 9804 to a bacterial pathogen of rice, *Xoc*. The difference between 9804-*Rxo1 *and 9804 in this aspect revealed some interesting aspects on the molecular mechanisms underlying the non-host resistance of rice mediated by the maize R gene, *Rxo1*.

First, the non-host maize R gene, *Rxo1*, was able to induce the differential expression of large numbers and diverse categories of genes in the heterologous rice plants, 9804-*Rxo1*, but not in 9804 under the infection of *Xoc*. This clearly indicates that the maize R gene was involved in the early steps of the interaction between rice and *Xoc*.

Second, consistent with the HR phenotype of 9804-*Rxo1 *to *Xoc*, our results that numerous genes associated with each step of HR were specifically up-regulated in the infected 9804-*Rxo1*. In particular, the specific group of WRKY TF genes induced only in 9804-*Rxo1 *are known to be involved in HR of plants [21,27]. In particular, 6 AP2 TF genes were uniquely differentially expressed in 9804-*Rxo1*, suggesting their involvement in the HR of the transgenic rice plants mediated by *Rxo1*. Thus, our results strongly suggest some common molecular mechanism(s) of *Rxo1 *and typical host R genes in mediating HR in rice. However, we noted that there was no change in the expression level of *NDR1 *and *PBS2 *in the infected 9804-*Rxo1 *plants, which are known to be regulated by typical CC-NBS-LRR type of R genes in the interaction between plant and pathogen [28-32]. Although *Rxo1 *belongs to the group of NBS-LRR [10], how its product recognizes effector (s) of *Xoc *and triggers signal transduction cascade leading to HR remains a mystery.

Third, a large set of genes encoding PPR and RRM proteins were uniquely up-regulated in 9804-*Rxo1 *by *Xoc*, but not in 9804, suggesting that the post-transcriptional modification might be an important feature in the *Rxo1 *mediated HR in the heterologous rice plants. PPR and RRM proteins are reportedly involved in a wide range of different post-transcriptional processes in plant organelles [33-35] and may also affect nuclear gene expression by plastid to nucleus signaling pathway [36]. The presence of a common G-box, AACTGG, within the cis-element in the promoter regions of these PPR genes strongly suggests these PPR genes may have been involved in the JA mediated disease responses [14]. It is generally accepted that SA plays a major role in activating defenses against biotrophic pathogens, whereas JA and ET are usually associated with defenses against necrotrophic pathogen attacks [37]. Thus, the cross-talk between these two defense signaling pathways is important in plant defenses system to specific pathogens [38,39]. In this respect, our results suggested that *Rxo1 *could activate the gene networks of some basal defense signaling pathways in rice, including both ET and JA-dependent signaling pathways, even though how *Rxo1 *mediated the complex signaling pathways leading to HR of rice to *Xoc *remains to be elucidated.

## Conclusions

The molecular mechanisms underlying HR of rice to its bacterial pathogen, *Xoc *mediated by a non-host maize R gene, *Rxo1 *were investigated using a microarray experiment and a pair of transgenic and non-transgenic rice lines. Our results indicated that *Rxo1 *appeared to function in the very early step of the interaction between rice and *Xoc*, and could specifically activate large numbers of genes involved in signaling pathways leading to HR and some basal defensive pathways such as SA and ET pathways. In the former case, *Rxo1 *appeared to differ from the typical host *R *genes in that it could lead to HR without activating *NDR1*. In the latter cases, *Rxo1 *was able to induce a unique group of set of WRKY TF genes and a large set of genes encoding PPR proteins that share the same G-box in their promoter regions with possible functions in post-transcriptional regulation. Some key genes that function in the downstream of *Rxo1 *were identified, including *OsNPR1 *and *OsPR1*. Thus, our results elucidated some interesting aspects on the molecular mechanism of the non-host resistance of rice mediated by *Rxo1 *and provided useful information to understand on the evolution of plant resistance genes.

## Methods

### Plant materials and artificial inoculation

A japonica rice variety, 9804, was used as the recipient to transform the maize *Rxo1 *gene in our transformation experiment using the standard *Agrobacterium *mediated transformation system, as described previously [40]. A homozygous T_4 _line containing a single copy of *Rxo1 *was obtained from the transgenic 9804 plants and designated as 9804-*Rxo1*. The 9804-*Rxo1 *line exhibited a higher level of resistance to *Xoc *strains, as reported previously [40]. Seeds of 9804-*Rxo1 *and 9804 (check) were sown in the seedling nursery, and 30-days-old seedlings of each line were transplanted into an isolated screening house at a spacing of 25 × 20 cm with 1 seedling per hill in the Fujian Academy of Agricultural Sciences.

A virulent *Xoc *strain, FJR5, collected from the rice field in the Fujian Province, China, was used in this study. The isolate was incubated on peptone sucrose agar (PSA) at 30°C for 3 days and the inoculum was prepared by suspending the bacterial mass in sterile water to a concentration of ~10^8 ^cells/ml. At the booting stage, flag leaves of of the 9801-*Rxo1 *and 9804 plants were inoculated using FJR5 by a pin pricked method [41], and each flag leaf was pricked at the tip, middle and end part, respectively.

### Histochemical staining for lignin

To determine the difference between 9804-*Rxo1 *and 9804 in the presence of lignin in leaf tissues resulting from *Xoc *infection, which is known to be an important characteristic of plant HR, histochemical staining for detecting lignin was carried out according to the method described by Gay and Tuzun [42]. Inoculated leaves for 9804-*Rxo1 *and 9804 were stained for lignin using 10% phloroglucinol treated with concentrated HCl for 2 days post-inoculation (dpi). Lignin stained a red color, which darkened to black with increasing quantities. Twenty leaves were stained for each treatment per variety per experimental replication. The stained leaves were observed under a light microscope and recorded photographically.

### RNA extraction and processing of microarray analysis

All inoculated flag leaves from three plants of 9804-*Rxo1 *and 9804 were collected two days post-inoculation (dpi), and the flag leaves from three non-inoculated check plants of 9804-*Rxo1 *and 9804 were also sampled at the same time point as control. The collected leaves were immediately frozen in liquid nitrogen, and then kept at -70°C. Briefly, the total RNA was extracted from each frozen leaf sample using the TRIZOL reagent according to the instruction, and then purified and concentrated using the Qiagen RNeasy MinElute Cleanup kit.

RNA was quality-checked and quantified using a Bioanalyzer 2100 (Agilent Technologies, Cheadle, UK). RNA from the three independent replicate samples of each treatment were used for hybridization to the Affymetrix Genechips^® ^Rice Genome Array chips, which contain probes to query 51,279 transcripts from two rice cultivars, including 48,564 japonica transcripts and 1,260 indica transcripts from Affymetrix (Santa Clara, CA). Preparation of cDNA, cRNA, hybridization to the arrays and quality control checks were carried out at the Company of CapitalBio Corporation, Beijing. The scanned images of the hybridization chips were firstly examined by visual inspection and then processed to generate raw data saved as CEL files. The whole set of microarray data has been have been deposited in NCBI's Gene Expression Omnibus and can be freely accessed through GEO Series number GSE19239.

### Analysis of microarray data

The GeneChip Operating Software (GCOS1.4) was used to analyze the raw scanned image data in the CEL files. The normalization of all arrays was performed in a global scaling procedure by the dChip software. Then, the SAM (Significant Analysis of Microarray) software was applied to identify differentially regulated genes (DRGs) between samples using the two classes unpaired method based on the empirical criterion of more than two-fold change and significant *q *value (FDR adjusted *P *value) less than 0.05. A list of known or unknown genes was generated by performing a BLASTn search http://www.ncbi.nlm.nih.gov/blast/Blast.cgi from the Affymetrix website. The putative function of each gene corresponding to the probe set on the chip was predicted by the Affymetrix annotation combined with the TIGR definition and NCBI database. The cis-element analysis for specific sets of DRGs of interest was performed as described by Pavesi et al [43].

### Expression validation using RT-PCR and quantitative real time PCR

In order to validate the results of the microarray experiment, a subset of *Xoc *induced DRGs were verified by RT-PCR. An independent set of rice plants of both 9804-*Rxo1 *and 9804 were grown and infected with *Xoc *as those prepared for microarray analysis previously. Three replicate leaf samples were taken for each treatment at each time point. First-strand cDNA was synthesized from 2 μg of total RNA using the first strand cDNA synthesis kit (Takara) and subsequently purified using a PCR purification kit (Takara) according to the manufacturer's instructions.

Quantitative real time PCR was followed the methods described by Swarbrick *et al*. [16]. The sequence of each gene was obtained from the TIGR rice database, and the exonic sequences from each gene were used for designing the primers by Primer 3 software http://frodo.wi.mit.edu/ [see Additional file 7]. The leaf samples were collected at 2, 3, 5 and 7 dpi, respectively. RNA samples from three independent replicates for each treatment were pooled before synthesis of cDNA. Expression values of the microarray experiment were normalized to minimize the differences in cDNA inputs using the parallel reactions against the amplified *Actin *gene product (Os03g50890).

## Authors' contributions

YZ designed the experiments and drafted the manuscript. MX executed all experiments. MZ, LZ and XX performed the phenotypic experiment and inoculation experiments of the microarray experiment. BF designed the microarray experiments, performed the data analyses of microarray data and revised the manuscript. ZL revised the final version of the manuscript. All authors have read and approved the final manuscript.

## Supplementary Material

Additional file 1 **List of commonly regulated genes in the 9804-Rxo1 and 9804 under the infection of *Xanthomonas oryzae pv. Oryzicola***. Excel file contains all commonly regulated genes in the 9804-Rxo1 and 9804 under the infection of *Xanthomonas oryzae pv. Oryzicola*. Click here for file

Additional file 2 **List of the TF genes differentially regulated by *Xanthomonas oryzae pv. oryzicola *in 9804-*Rxo1***. Excel file contains the transcription factor genes regulated by *Xoc *in 9804-*Rxo1*. Click here for file

Additional file 3 **List of the TF genes differentially regulated by *Xanthomonas oryzae pv. oryzicola *in 9804**. Excel file contains the transcription factor genes regulated by *Xoc *in 9804. Click here for file

Additional file 4 **List of 82 DRGs functionally classified as post-transcriptional regulation in 9804-*Rxo1 *under the infection of *Xoc***. Excel file contains all 82 differentially regulated genes classified as post-transcriptional regulation in 9804-*Rxo1 *under the infection of *Xoc* Click here for file

Additional file 5 **The logos for the three motif sequences of AACTGGAC, GAAACTGG and AACTGG identified in the upstream of the 65 specifically up-regulated PPR genes in 9804-*Rxo1 *induced by *Xoc***. Three logos for the motif sequences identified in the upstream of the specifically up-regulated PPR genes in 9804-*Rxo1* Click here for file

Additional file 6 **Genes involved in calcium ion fluxes up-regulated in infected 9804-*Rxo1***. Excel file contains the list of genes involved in calcium ion fluxes up-regulated in infected 9804-*Rxo1* Click here for file

Additional file 7 **List of primers for quantitative PCR**. Excel file listed all primer sequences used for quantitative PCR in this study Click here for file
