# Supplementary material for: Genome-wide gene responses in a transgenic rice line carrying the maize resistance gene Rxo1 to the rice bacterial streak pathogen, Xanthomonas oryzae pv. oryzicola
Source: BMC Genomics. 2010 Feb 1;11:78. doi: 10.1186/1471-2164-11-78 (PMC2824728; doi:10.1186/1471-2164-11-78)
Supplement: Additional file 5 — The logos for the three motif sequences of AACTGGAC, GAAACTGG and AACTGG identified in the upstream of the 65 specifically up-regulated PPR genes in 9804-Rxo1 induced by Xoc. Three logos for the motif sequences identified in the upstream of the specifically up-regulated PPR genes in 9804-Rxo1 [file 1471-2164-11-78-S5.PPT]

## Slide 1
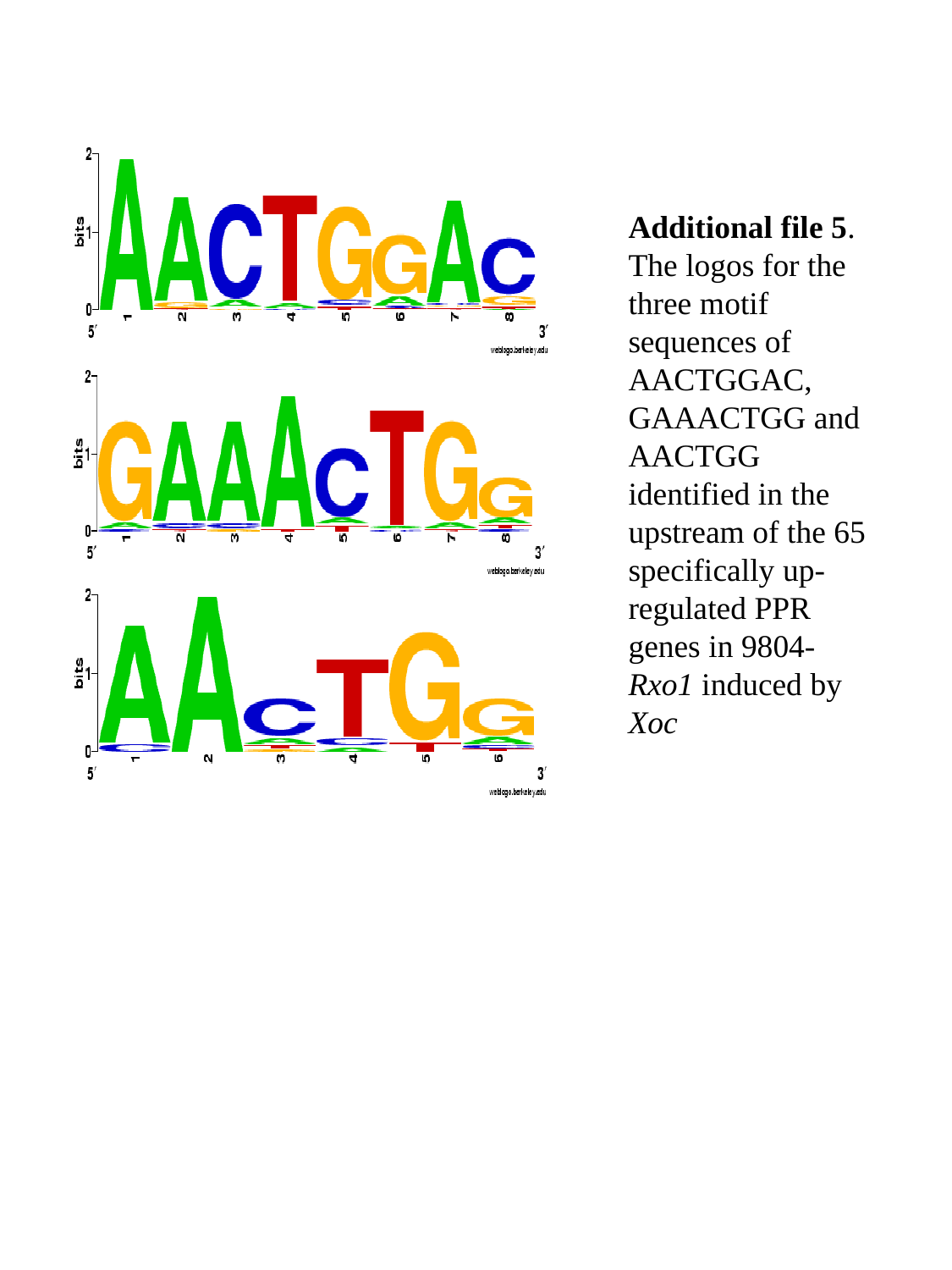

Additional file 5. The logos for the three motif sequences of AACTGGAC, GAAACTGG and AACTGG identified in the upstream of the 65 specifically up-regulated PPR genes in 9804-Rxo1 induced by Xoc
